# Using Social Media to Recruit Participants in Health Care Research: Case Study

**DOI:** 10.2196/51751

**Published:** 2024-10-11

**Authors:** Amy L Wright, Ysabella Jayne Willett, Era Mae Ferron, Vithusa Kumarasamy, Sarah M Lem, Ossaid Ahmed

**Affiliations:** 1 Lawrence Bloomberg Faculty of Nursing University of Toronto Toronto, ON Canada

**Keywords:** social media, qualitative methods, recruitment strategies, healthcare research, digital health, internet

## Abstract

This paper presents a case study describing the use of social media, specifically Facebook and Instagram, as a valuable tool for recruiting participants in community-engaged health care studies. Drawing on the experiences of our team during a qualitative study aiming to understand the needs of Indigenous fathers and Two-Spirit parents as they transition to parenthood, we offer an in-depth exploration of our social media recruitment strategy. This strategy encompasses deliberate content creation and online engagement with local Indigenous community organizations and people. Through the implementation of this recruitment strategy, we successfully recruited 18 Indigenous fathers and 4 Two-Spirit parents to our community-engaged project. We learned that social media can be used to enhance recruitment by building community trust, engagement, tailored content for specific audiences, and adaptive strategies guided by data metrics provided by social media platforms. Our journey included several challenges, such as dealing with fraudulent participants, navigating budget and resource constraints, and facing recruitment limitations, which we also describe in detail. Our paper provides essential insights for researchers considering the use of social media as a recruitment tool but we are unsure of how to begin. Health care researchers may find our experience and recommendations helpful for developing and implementing their own effective social media recruitment strategy. Meanwhile, sharing our experience contributes to the broader understanding of the role of social media in participant recruitment.

## Introduction

Social media refers to online platforms and technologies that facilitate the creation, sharing, and exchange of user-generated content and enables interactions and connections between individuals and groups in an online space [1]. These platforms often support various forms of content, including text, images, videos, and links, allowing users to communicate, share information, and build online communities. Social media, with its current estimate of 3.8 billion active users worldwide [1,2], has transformed the way researchers connect with potential study participants. In the era of digital connectivity, social media stands as a powerful tool, offering an opportunity for researchers to harness this digital landscape for participant recruitment beyond traditional communication methods, such as word-of-mouth, posters, newspaper advertisements, and direct mailing [1,3]. Facebook, Instagram, and X (formerly Twitter) stand as prominent social media platforms, collectively hosting billions of monthly users, and have gained popularity among researchers for participant recruitment [4-6]. Facebook allows users to create individual profiles and organizational pages to foster community building around common interests [7]. In contrast, Instagram offers a visual communication stream, with a primary focus on photos, graphics, and videos [8], catering to younger users with a preference for visual content [9]. Like Facebook and Instagram, X allows users to share and discover short text messages, as well as various multimedia content, including photos, videos, and live streams [10]. In this paper, we focus our discussion on the 2 social media platforms that we used in our study: Facebook and Instagram. We chose not to use X due to limits on time and the funding necessary to implement our social media recruitment strategy across more than 2 platforms.

### Benefits of Using Social Media for Recruitment

The versatility of social media has positioned it as a valuable resource for researchers to disseminate study information to the public, generate interest, and enhance recruitment efforts [11-13]. The role of social media in health care research has grown considerably over the years, serving as a platform to improve research literacy and individuals’ understanding of ongoing research studies [9,11]. Using social media for participant recruitment in health care–related studies has several benefits. First, it presents a cost-effective approach to recruitment [13,14], making it particularly appealing to researchers with limited budgets. Second, social media offers extensive accessibility, reaching a diverse global audience using various electronic devices [4,13,15], and allowing for anonymous interaction by users [4,16,17]. It has been found to be highly effective in connecting with adolescents and young adults, who are active users [9]. Third, it aids in engaging individuals who have historically been difficult to recruit due to stigmatization, social inequities, low disease prevalence, or disabilities [4,9,16,18-21]. Facebook, in particular, has been recognized for its success in reaching underrepresented communities, allowing researchers to connect with individuals based on demographics, geographical location, interests, and behaviors [3,5,22]. Lastly, social media platforms can support researchers in reaching their desired sample sizes, a crucial advantage for studies requiring large participant populations [3].

Despite these benefits, there are some challenges worth noting. First, using social media for recruitment requires users to have electronic devices with access to the internet, so recruitment using only social media may exclude those without this access [4,5]. Second, there is an increased risk of fraudulent participants, threatening data integrity [16,17,19-21,23]. This concern will be outlined later in detail.

Our paper presents insights gained from using Facebook and Instagram to recruit participants for phase 1 of a 3-phased community-engaged research study, Fathers of the Next Generation (FOTNG). By using this example as a case study with which to illustrate our use of social media for participant recruitment and its associated risks and benefits, we aim to provide researchers with key insights to consider to effectively develop and implement this type of recruitment strategy. For definitions of common social media terminology used in Facebook and Instagram, please see Multimedia Appendix 1.

### Case Study: FOTNG

The FOTNG is a 3-year, 3-phase project, funded by the Canadian Institutes of Health Research. The project focuses on understanding the unique needs of self-identifying First Nations, Métis, and Inuit fathers and Two-Spirit parents during the transition to parenthood to build a community-derived parenting program that addresses their needs. Recognizing the diversity of Indigenous Peoples, we will use the term Indigenous to refer to more than one cultural group, and use the specific culture or Nation whenever possible. This study received ethics approval from the Six Nations Council Research Ethics Committee and the University of Toronto Research Ethics Committee. The project is led by a steering committee of representatives from Indigenous-led organizations within Southern Ontario, Canada, Indigenous service providers, parents, and Indigenous and non-Indigenous researchers of settler-European ancestry. The first phase of the project involves a qualitative study, which commenced participant recruitment in September 2022 and will be the focus of our discussion here. Phase 1 included one-on-one interviews with self-identifying Indigenous fathers, Two-Spirit parents, and service providers. Parent participants were eligible to participate if they identified as an Indigenous father (or father-to-be), or Two-Spirit parent (or parent-to-be), and had a child aged younger than 3 years or were expecting a baby, and lived in Southern Ontario, Canada. Service providers were eligible to participate if they provided parenting programs or services to Indigenous fathers or Two-Spirit parents in the same region. At the time of publication, we have recruited 18 Indigenous fathers, 4 Two-Spirit parents, and 23 service providers, and recruitment for Two-Spirit parents is ongoing. Although this phase also included the recruitment of service providers, we will focus our case study on the recruitment strategy we used for fathers and Two-Spirit parents, as service providers were primarily recruited through word-of-mouth by our community partners and steering committee.

Our recruitment strategy was devised by the lead author and steering committee and supported by a project coordinator and 2 research assistants, and included numerous strategies including traditional approaches: word-of-mouth, distributing digital and hard copies of flyers, posting flyers and other information on the project website, attending community events, in addition to a comprehensive online social media communication strategy. As our project is community-led by Indigenous organizations and community members, we collectively agreed it was important to use our social media platforms to not only recruit participants to participate in our project but to also build awareness among Indigenous and non-Indigenous people about Indigenous cultures and priorities, inform the public about our study and ongoing activities and findings, and, most importantly, facilitate trust and building relationships with Indigenous community members and organizations.

### Social Media Communication Strategy: Initial Steps

Any researcher seeking to use social media to recruit participants must first have a social media account. After creating an account for our project, we established our presence on both Facebook and Instagram by following the accounts of Indigenous organizations, relevant health and wellness groups, Indigenous activists, educators, and other established Indigenous community members. Following these accounts helped us to elicit interest in our account through other users by sharing their content on our page. Before we began to create our own content, we developed a logo and short project name (FOTNG) to support our study’s *brand recognition.* We developed a project logo with support from a graphic designer and the steering committee. The project logo and its associated color palette provided a standardized and appealing visual that was crucial for establishing our project’s recognition within our social media account profiles and across posts [22].

### Content Creation

**Figure 1:**
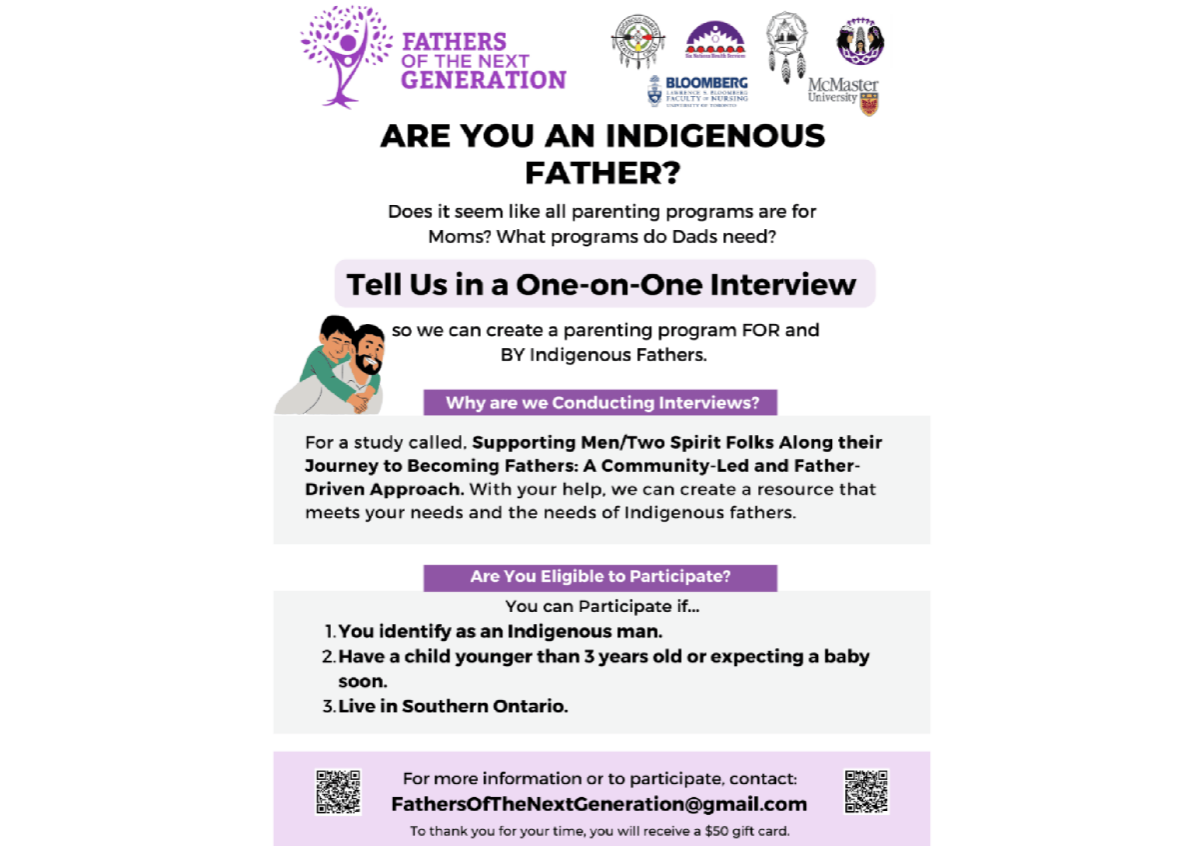
Recruitment flyer for Indigenous fathers. Note: the name and phone number of the research coordinator have been removed due to privacy considerations. Text, images, and graphics were created using Canva.

**Figure 2:**
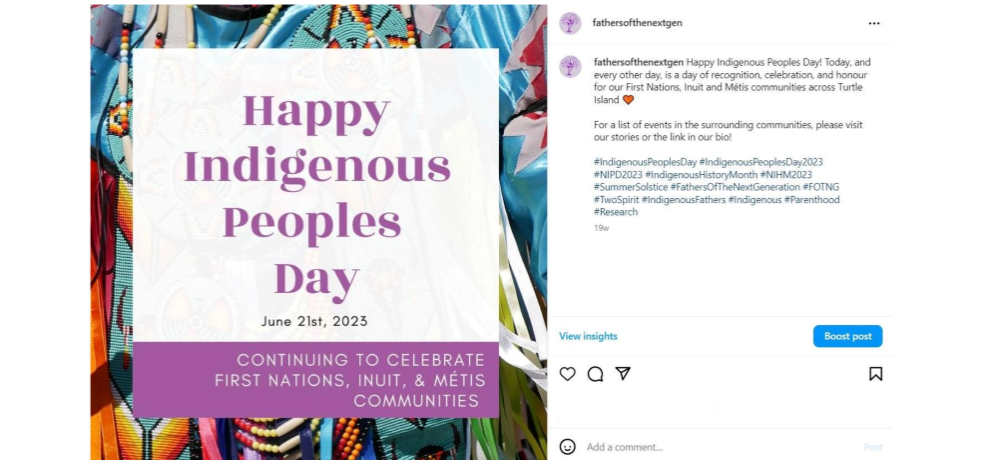
Single image post commemorating Indigenous Peoples Day [24]. The right side of the figure displays the accompanying caption and indication of the number of likes and comments. Text, images, and graphics were created using Canva.

**Figure 3:**
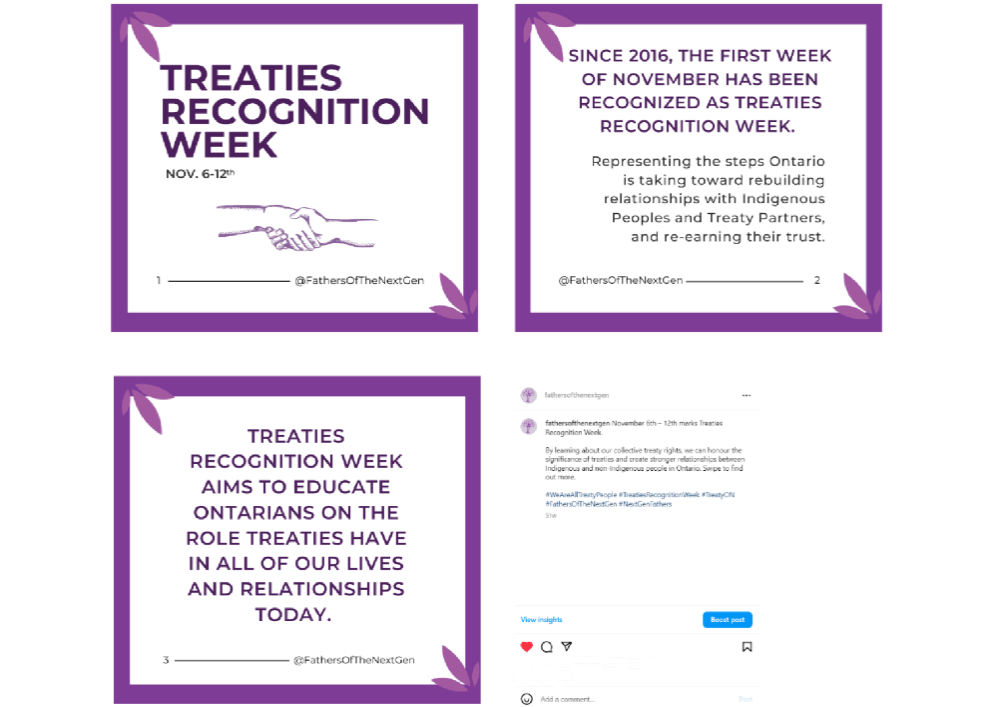
Carousel post for Treaties Recognition Week [25], an important Indigenous event celebrated every November in the province of Ontario, Canada, and accompanying caption. Text, images, and graphics were created using Canva.

Our content strategy was guided by three objectives: (1) to create awareness about Indigenous wellness, including information relevant to this study (parenting), family health and wellness, and Indigenous holidays and observances; (2) to recruit Indigenous participants who met our eligibility criteria; and (3) to communicate study activities and findings. We posted our recruitment flyer, which contained the purpose of this study, eligibility requirements, incentive information, and research coordinator contact information (see Figure 1). We used single images (Figure 2 [24]), carousels (several images in a single post; Figure 3 [25]), graphics, and reels or videos to share information. We reshared content multiple times to reach new people [26], and to foster engagement and support among our followers. Initially, we posted content about the project, such as this study’s purpose, objectives, and phases, and once we covered relevant project details, we shifted our focus to promoting participant recruitment and celebrating and promoting health and wellness among parents, young children, families, and communities.

To ensure our content was informative and visually appealing, our team carefully conducted thorough research and sourced credible information, and included graphics found using the platform Canva. Captions were used to summarize information not depicted in the visual content and typically included a summary, contact details, a link to the FOTNG website, and relevant hashtags [11,28]. Hashtags are links to other relevant social media topics (eg, #FOTNG), and by adding these to our posts, we hoped to promote the engagement of the followers interested in those topics with our content [29]. Finally, we were deliberate in our choice of language, aiming to maximize end user engagement by keeping content simple, concise, and at an 8th-grade reading level or a Gunning Fog Index score of less than eight [30,31].

Further, 2 research assistants managed content creation and posting to the FOTNG social media accounts. They met monthly to brainstorm ideas and adjust their approach based on social media data metrics provided through the platforms. For instance, we initially posted a 58-second recruitment video which reached 123 user accounts on Facebook. To increase the number of people viewing our recruitment video content, we condensed the video to a 21-second reel, which increased our engagement 12-fold, reaching 1628 user accounts. Our experience is supported by the literature indicating that shorter videos (less than 60 seconds) are more engaging [32,33], also reflecting end user preference for quick access to information in today’s fast-paced world [34].

### Posting Timing and Frequency

Before we were granted access to special data metrics detailing network growth, top locations and age range of followers, and times of most activity on each platform, we experimented with posting at various times of day and frequencies, to identify audience engagement trends. Once our Instagram page reached over 100 followers, we were provided access to special insights and data metric reports via the platform, which we used to adjust our posting schedule to align with peak user activity on Instagram, primarily between 12 PM and 6 PM EST, on Tuesdays and Fridays. Additional posts occurred every other week as part of our monthly social media plan. For Facebook, our follower count remained below the 100 users required to gain access to special data metrics, so we were unable to use these data to inform our approach. Instead, we mirrored our Facebook posting schedule to match our Instagram metrics, using the same peak times and preferred days of the week [12,35].

Notably, posts of our recruitment flyer generated the highest engagement concerning views, shares, impressions, and accounts reached compared to other posted content. This aligns with the experiences of Luarn et al [36], who found that posts advertising tangible benefits (such as compensation and honoraria) were more engaging than posts without. Additionally, we observed increased engagement on both Facebook and Instagram when our content was posted more frequently. For example, months with an average of 3 to 5 posts per week garnered greater impressions and engagement than those with only 2 to 3 posts per week. Recognizing that posting too frequently can diminish end user interest and engagement, we sought to balance our posting schedule to maximize interaction [37].

### Connecting With Respected Community Members and Organizations

#### Overview

We prioritized building relationships with other relevant account holders, to support the engagement of our followers with their content by sharing their posts on our platforms, as well as to promote engagement with potential research participants, by their sharing our posts on their platforms. These account holders included Indigenous organizations, parenting and early childhood education groups, and popular leaders within Indigenous communities in Southern Ontario. To establish connections with these accounts, we sent private messages via the platform requesting they share our recruitment flyer with their followers. In turn, we shared their content on our sites. We aimed to foster trust and build online relationships with the account holders and their followers by promoting one another’s content. Additionally, we asked our partner organizations and steering committee members to share our content on their personal and organization’s social media accounts. As trusted sources of information, their sharing of our content enhanced the credibility of our accounts and instilled a stronger sense of confidence among eligible participants who contacted us to express their interest in participating [12,38].

#### Using Data Metrics

By analyzing data metrics, we recognized better engagement with our Instagram account than with our Facebook account. This was made evident by the number of likes, comments, and reactions to posts and stories, and followers, as well as the additional special data metrics provided by Instagram. Further, 1 reason for the difference in engagement between the 2 platforms may be the increased popularity of Instagram among adolescents and young adults, compared with Facebook [39]. While Facebook has been identified as the most widely used social media platform among researchers, its use is declining among younger users [2,9]. As a result, Facebook may not have been as appealing to our target audience, which primarily consisted of parents, generally aged younger than 40 years [40]. Despite this, more study participants expressed that they became aware of the FOTNG study through our Facebook page rather than our Instagram site.

How we delivered content also impacted user engagement. Data metrics on both Facebook and Instagram showed that content delivered in the form of a carousel performed better than single-image posts, and visually appealing content garnered more attention from our target audience, regardless of the day of the week or time the content was posted. In addition, we found that posts containing the faces of actual people had more views in comparison to posts with text-only, cartoon images, or other graphic variations. For instance, our World Smile Day post on Facebook, which consisted of actual human faces, reached more than 10 times the number of accounts than our text-only image posted 10 days later which outlined the FOTNG study framework. This is in line with the findings by Cyhlarova et al [38], who found that more personable recruitment methods are more effective. Similarly, posting on and about Indigenous-specific national holidays or observances performed significantly better than posts about Canadian national holidays or observances. As an illustration, during Treaties Recognition Week (Figure 3 [25]), we posted content that detailed the history and importance of acknowledging treaties in our local area. This post reached nearly 5 times the number of accounts on Facebook and nearly double the number of Instagram accounts when compared to our World Kindness Day post, which was published 7 days later. Our experience suggests that content specific to the target audience’s cultural, spiritual, and historical needs and interests may generate more engagement and interactions than other content. As explained by Sutherland [41], creating appealing content that is tailored to a target audience’s interests can foster an online community with which end users can connect and interact, generating greater engagement.

#### Time Invested by the Research Team

Gaining attention, credibility, and followers on Facebook and Instagram involved a significant amount of time and effort on behalf of our research team. Specifically, devising and implementing our social media recruitment plan involved: (1) brainstorming, researching, and sourcing appropriate topics; (2) creating clear, concise, engaging, and culturally appropriate content; (3) sourcing appropriate visuals; (4) prescheduling content; and (5) analyzing data metrics [41]. While these efforts were necessary to present relevant, worthwhile content that our followers wanted to engage with [41], it required 2 research assistants to spend a total of approximately 10 hours per week over 3 months to develop and execute our social media recruitment strategy to recruit 22 parent participants. Our experience aligns with Sutherland [41] who found that teams need to dedicate extensive time to researching their targeted audience and creating evidence-based social media strategies that can guide actions and decisions. Failing to allocate an adequate amount of time and funding to content creation can result in posts containing incorrect information or poor use of images, potentially damaging the account’s (and study’s) reputation.

## Discussion: Lessons Learned

Thus far we have described our approach to creating and implementing an online recruitment strategy using Facebook and Instagram social media platforms. This approach has been successful in creating community engagement with local Indigenous organizations, parent and child-focused organizations, and Indigenous community members. Combined with other more traditional recruitment strategies, such as distributing and posting hard copies of flyers, attending events, and word of mouth, we have achieved our recruitment goals. Throughout our use of online recruitment using social media, we have realized many benefits as well as challenges, which we outline next. These are particularly salient for researchers considering using online recruitment strategies, to design a thoughtful and effective, yet informed and cautious approach.

### Benefits

#### Community Trust and Engagement

One of the benefits of using social media for participant recruitment is engaging and building trust with the community from which our target participants are members. Our recruitment strategy was intentionally designed to foster a safe and trustworthy online space for Indigenous families. By not only sharing content directly related to our study, but also content around Indigenous-specific cultural holidays and observances, and information about family health and parenting, our efforts facilitated public engagement and trust in the project and demonstrated our sincere desire to support Indigenous communities [41]. The trust of local organizations in our project became evident as several Indigenous-led organizations shared our recruitment materials with their followers. This collaborative effort resulted in individuals reaching out to participate in this study. While we did not systematically collect data on how participants learned about our study, many individuals voluntarily informed us during the screening process that they learned about our research through recruitment flyers posted on Indigenous community organizations’ social media accounts.

#### Tailored Content

Another benefit that social media has to offer researchers is the distribution of tailored content through diverse formats [13]. Facebook and Instagram allowed us to disseminate our original recruitment flyer as well as iterations of it that were more visually appealing, in addition to standalone posts, carousels, and reels or videos of varying lengths. Data metrics from each platform also showed us that by tailoring our content to cultural, spiritual, and historical interests, and featuring real people’s faces (eg, fathers and children), we significantly improved engagement, as they emphasized relatability [38,41].

#### Adaptability through Data Metrics

Data metric analyses are another benefit of leveraging social media to recruit participants as the data informs researchers of the effectiveness of their content and can be used to quickly revise one’s approach to better meet the engagement activities of users. For example, after reflecting on the analytic data, we were able to quickly shorten our recruitment video from 58 to 21 seconds increasing our engagement by 12 times the number of followers.

### Challenges

Using Facebook and Instagram for recruitment was not without significant challenges, however. Fraudulent participants, budget constraints, inaccurate data metrics, and user access are real concerns to be thoughtfully considered before initiating a recruitment strategy using social media.

#### Fraudulent Participants

First, the presence of fraudulent participants, defined as ineligible persons or computer bots designed to pose as real people in a research study [17], is a known pervasive problem in health care studies where online recruitment methods are used [16,42]. As our recruitment materials initially mentioned a CAD $50 (US $37) gift card honorarium as an incentive for participation, some individuals (or bots) were motivated to falsify their eligibility and participate in this study in hopes of receiving the honorarium [20,43,44]. After discovering a fraudulent participant had passed our email screening process undetected, the research team quickly modified the screening procedures to mitigate the enrollment of fraudulent participants. The modified process included screening over the phone instead of by email, allowing the project coordinator to interpret an individual’s eligibility by gauging the ease with which they provided answers to screening questions. Realizing that outlining this study’s specific inclusion criteria in our social media posts had made it easy for fraudulent participants to answer our screening questions, we removed many of these details from our posts and redesigned our questions to be challenging for ineligible people to answer. For instance, we changed an original screening question from “Do you live in Southern Ontario” to “Where do you live?” If an individual was unable to answer one of our screening questions, they were deemed ineligible to participate. Given that we did not use a more targeted online recruitment approach (eg, paid advertisements) to reach eligible individuals, we inevitably reached a large pool of people, any one of whom might choose to participate fraudulently. This put us at risk of collecting fraudulent data [21] and resulted in a great deal of stress and wasted time for the research team.

Researchers planning to leverage social media for recruitment purposes should be aware of the growing threat of fraudulent participants and incorporate strategies to prevent its occurrence and mitigate its impact. Lawlor et al [45] present a structured approach for researchers to both prevent and detect fraudulent participants in their studies called the REAL framework. This method consists of 4 key stages. First, researchers are prompted to reflect on the vulnerabilities within their recruitment plan, considering the survey design elements in place to minimize fraudulent entries. Second, they establish expectations by identifying typical data patterns and anomalies they might encounter. In the third stage, researchers analyze collected data to check for any deviations from these expectations. Finally, the framework emphasizes transparency through the establishment of criteria to label and exclude potentially fraudulent responses. Creating a recruitment strategy using the REAL framework can support researchers in anticipating and mitigating the threat of fraudulent participants.

#### Budget and Resource Constraints

A second challenge was the substantial financial investment required to create and implement a social media communication plan and recruitment strategy [2]. Each social media post necessitated several hours of work, from researching topics to finding appealing graphics and writing captions. To maintain a presence in our followers’ social media feeds, we needed to create frequent and original content, which is also time-consuming [12]. This can represent a significant challenge for researchers, particularly if time and budget are limited. Researchers must carefully consider whether they have the necessary personnel and funds to use an online recruitment strategy, especially without a guaranteed achievement of the desired sample size [46].

#### Data Metrics Versus Participant Feedback

A third challenge involved inaccurate interpretation of the data metrics provided by the social media platforms. In our project, we learned that metrics can sometimes misrepresent the actual reach of content. For instance, our metrics on Facebook indicated limited engagement with our target audience compared to Instagram, yet many study participants reported discovering our study through Facebook, despite not following our page. This discrepancy underscores the importance of asking participants directly about how they learned about this study to gain a more accurate understanding of whether the recruitment strategies being used are effective.

### Access Limitations

Finally, although social media provided us with access to a diverse and massive set of individuals, our social media recruitment efforts were likely limited to people with access to an electronic device and the internet, which may have introduced sampling biases [12]. To mitigate this limitation, we encouraged study participants to share information about this study within their own social networks [47] and asked our partner organizations and steering committee members to do the same. We also used additional traditional means of recruitment, such as distributing hard copies of the flyer and attending in-person and online events to share our study. Using traditional methods and continuing to prioritize in-person recruitment strategies was important to our steering committee, which prioritizes building relationships with members of the communities we are working with throughout our project to inform the project design and implementation, give back to community members, and support knowledge dissemination efforts. Participants’ access to information about study participation is important for all researchers to consider, particularly if recruiting using social media will introduce critical sampling biases that may jeopardize study findings. For instance, using social media is likely not an effective way to recruit participants where internet access is low or absent, such as in some rural areas, or when participants are unlikely to use social media or own a device with internet access, such as young children. Researchers should carefully reflect on how their intended participants access information and make efforts to mitigate the risk of sampling biases in their recruitment strategies.

### Recommendations and Future Research

Given our experience developing and implementing an online recruitment strategy using popular social media platforms, we have several recommendations for researchers interested in using this approach, as well as areas that require further research.

#### Create an Online Recruitment Plan

First, researchers must make well-considered decisions about online recruitment, considering factors such as platform suitability and feasibility given this study’s objectives. A thoughtful plan is essential for guiding researchers in making informed choices about which platforms are most likely to yield successful results. For example, Macapagal et al [46] present the CAN-DO-IT model as a guide for researchers to use when using online recruitment methods, irrespective of platform. The model encompasses 7 iterative steps, from conceptualization to implementation and evaluation of a recruitment strategy. Researchers can support the growing body of literature on online recruitment strategies by writing about how they design their approaches, their experiences of effectiveness, and other lessons learned.

#### Fraudulent Participant Prevention Strategies

Second, due to the threat of fraudulent participants in online research [48], it is imperative for researchers to plan for and implement prevention strategies, recognizing that more than one strategy is often necessary to mitigate this risk. Several prevention and verification actions may be used, such as using CAPTCHA [49] in online surveys, refraining from including the existence of incentives in recruitment advertisements, advertising in specific social media groups, using paid advertisements, checking IP addresses, screening for eligibility in real time and on-camera, requesting that participants show government-issued identification, and asking questions only legitimate participants would be able to answer with ease [19-22,44,49-54]. The REAL framework described earlier, can be used as a structured approach to help researchers prevent and detect fraudulent participants in their studies [45].

Given the growing popularity of online recruitment methods in research, we recommend that future research be conducted to better understand the problem of fraudulent participants. This includes exploring study-related factors that attract fraudulent participants, and the development of mitigation, verification, and prevention strategies that researchers can use in conjunction with social media recruitment strategies to encourage study participation without the added risk of compromising data.

#### Optimal Resource Allocation

Third, it is crucial to plan for adequate financial resources for recruitment purposes when writing grant funding applications. While the initial set-up costs of using social media for recruitment are often more cost-effective compared to traditional methods [14,19,21], the true costs of a social media approach may manifest in the form of human resource capital and time. Unlike mailing where sending flyers is a 1-time or infrequent effort to reach the target audience, social media requires a sustained and consistent posting strategy to effectively leverage the platform’s abilities to engage potential participants [19,55]. This entails dedicating time and effort to curate captivating and eye-catching content to capture the attention of potential participants and prevent them from simply scrolling past your content [46]. While incentives may help solve this problem, it is imperative to recognize that they may also attract fraudulent participants. As a result, researchers must allocate additional time to verify participant identities as discussed earlier. This may not pose a significant challenge for studies with smaller sample sizes, however, with large-scale studies (eg, Ali et al [47]), the added time and resource requirements can substantially impact the overall costs and project feasibility. Overall, the production of compelling content can translate into an increased workload for research staff, in addition to the time needed to analyze performance metrics, which are essential for optimizing user engagement.

#### Build Trust and Credibility

Fourth, building trust and credibility is very important when using social media for recruitment, particularly in community-engaged research with Indigenous communities, with whom unethical research practices have understandably resulted in distrust of researchers and academia [4,56-60]. In our experience, the use of social media enabled us to introduce our project to organizations and people we might otherwise have not encountered using traditional methods. However, our efforts to build an authentic online presence with support from Indigenous-led organizations and community members were what significantly enhanced our success. Researchers would do well to build relationships with community members and organizations relevant to their project aims, who can then support participant recruitment by sharing opportunities with their followers. This in turn will increase the reach of a social media campaign to engage participants who are more likely to be eligible to participate, while simultaneously fostering trust and credibility of the project to the public [4].

#### Contributing to the Literature

Fifth, as online recruitment methods are constantly evolving, collaboration is necessary for researchers to share experiences, challenges, and recommendations that can support one another in developing effective recruitment strategies. Researchers should detail their recruitment approaches and experiences in their publications and presentations, to contribute to the growing body of knowledge regarding social media recruitment. This collective effort can help to improve the efficiency and effectiveness of social media recruitment strategies, and help to address challenges such as representative bias [12,47] and fraudulent participants [20].

### Final Thoughts

Our case study offers important insights into the use of social media as a recruitment tool for engaging Indigenous fathers and Two-Spirit parents, using a community-engaged research approach. We have detailed our approach from its initial creation, considerations for creating content, and how one might interpret and respond to user engagement metrics. While we experienced numerous benefits, we also outline several challenges of using social media for study recruitment which researchers should consider before developing their approach. Despite these challenges, our social media accounts were able to foster trust and build relationships with other Indigenous-led organizations and community members in the virtual space, which supported our study recruitment through their sharing about our study on their platforms and promoting our project. Building trusting relationships with community groups, while ethically imperative in conducting research with Indigenous communities, proved indispensable to our recruitment efforts, and cannot be replaced by social media alone. This comprehensive approach, encompassing both social media and community engagement, allowed us to make significant strides in achieving our recruitment goals.

### Limitations

Several limitations of our experience are worth discussing. First, our experiences using social media for health care research is limited to Facebook and Instagram. Recruiting via other platforms might have generated more interest in our study. Our approach has also limited our ability to compare the effectiveness of Facebook and Instagram for study recruitment to other social media platforms such as X. Second, we used the special data metrics we were granted access to from Instagram to guide our social media plan for both Facebook and Instagram. Although our followers continue to increase, obtaining access to the special data metrics for Facebook is necessary for devising a tailored plan for this platform. Finally, as we did not intend to test our recruitment strategies for effectiveness at the outset of our project, we did not specifically ask participants how they learned about this study. Researchers should consider collecting this data in all their studies from the outset so they can gauge the effectiveness of their recruitment strategies and adjust their approach as needed.

### Conclusion

Our case study provides researchers with a detailed guide on what to consider and how to design and implement a social media recruitment strategy. Our experience is derived from a community-engaged project with Indigenous communities in Southern Ontario and demonstrates our ability to create relationships in an online space, which in turn enhanced the effectiveness of our approach. While there are many benefits to recruiting using social media, researchers should be thoughtful in their approach to devising a communication strategy, using data metrics to enhance user engagement, while planning mitigation tactics for the risk of fraudulent participants. Finally, researchers can contribute to the ongoing discourse on social media recruitment strategies by writing about their experiences in publications, facilitating the advancement of the use of social media for participant recruitment.

## Appendix

Multimedia Appendix 1 Terms used by Facebook and Instagram.

## Abbreviations

FOTNG: Fathers of the Next Generation
