# Supplementary material for: Using Social Media to Recruit Participants in Health Care Research: Case Study
Source: J Med Internet Res. 2024 Oct 11;26:e51751. doi: 10.2196/51751 (PMC11512130; doi:10.2196/51751)
Supplement: Multimedia Appendix 1 [file jmir_v26i1e51751_app1.docx]

**Table S1**. Terms used by Facebook and Instagram.

| Term | Description |
| --- | --- |
| **Shared by Facebook and Instagram** | |
| **Metrics** |  |
|  | Digital data used to compare and track the impact of social media activity for its intended purpose.[3,11]  Includes impressions, reach, and engagement, as well as end user demographics. |
| **Impressions** |  |
|  | Number of times content is viewed by end users indicating how often content is being revisited.[5] |
| **Reach** |  |
|  | Total number of end users viewing content at least once.[3,5]  Does not include the content that is *revisited* by the same users. |
| **Engagement** |  |
|  | End user interaction with content (liking, commenting, or sharing).[5] |
| **Post** |  |
|  | Umbrella term used to describe either the act of publishing content to social media accounts or the published content itself.[36]  Can include text, photos, graphics. |
| **Feed** |  |
|  | A continuous page on social media platforms that displays a constantly updated list of content from accounts followed by the end user.  An algorithm is generally used to understand the content the end user most frequently engages with and, as a result, displays that content most frequently at the top of their feed. |
| **Story** |  |
|  | Subcategory of posts. Refers to the culmination of photos, text, and relatively short videos that remain on an account temporarily and disappear after 24 hours.[61] |
| **Hashtag** |  |
|  | Word or phrase that turns into a clickable link when preceded by the symbol #.[29] |
| **Unique to Instagram** | |
| **Highlights** |  |
|  | Allows account holder to pin a story to the top of their account profile within the first 24 hours of posting their story.  Followers can view highlights anytime by visiting the account profile. |
| **Reels** |  |
|  | Short video clips up to 90 seconds long that consist of videos and/or photos combined into one cohesive video.  Can consist of several shorter videos that are edited into a single post.  They are posted to an account profile and can be reviewed as they are on a continuous loop. [61] |
| **Grid** |  |
|  | Visual layout of an account profile where previous posts are arranged in a grid–like pattern.[61]  Formed by consecutively posted content. Serves as a preview of the user’s content, providing a cohesive and aesthetically pleasing display of posts as a whole. |
| **Carousel** |  |
|  | Refers to a single post containing up to 10 photos or videos.[63] |

This is a Multimedia Appendix to a full manuscript published in the J Med Internet Res. For full copyright and citation information see 51751 [https://www.jmir.org/2024/1/e51751/]

**Additional References**

61. Instagram (2024). About. URL: https://about.instagram.com/ [accessed 2024-10-10]

62. Dragomir, M. (2024). Instagram grid maker: How to plan the perfect feed in 2024. Planable. URL: https://planable.io/blog/instagram-grid/ [accessed 2024-10-10]

63. Instagram (2024). Share a post with multiple photos or videos on Instagram. URL: https://help.instagram.com/269314186824048/?helpref=related_articles [accessed 2024-10-10]
